# The impact of citicoline on brain mitochondrial dysfunction induced in rats after head irradiation

**DOI:** 10.1038/s41598-025-11098-4

**Published:** 2025-07-15

**Authors:** Nahed Abdel-Aziz, Fatma Rabia Algeda, Shereen M. Shedid

**Affiliations:** Radiation Biology Research Department, National Center for Radiation Research & Technology, Egyptian Atomic Energy Authority, P.O. Box 29, Nasr City, Cairo Egypt

**Keywords:** γ-Radiation, Citicoline, Mitochondria, Oxidative stress, Biochemistry, Physiology

## Abstract

Head irradiation is a common treatment for brain cancer; however, it can cause side effects in healthy brain tissue. This study aimed to test whether citicoline administration modulates radiation-induced brain mitochondrial dysfunction in rats. The head of the animal was exposed to 10 Gy γ-radiation. Citicoline (300 mg/kg body weight/day) was administered intraperitoneally for four weeks after irradiation. Some biochemical changes related to mitochondrial function in brain tissue were studied. The results showed that citicoline administration after head irradiation reduced oxidative stress, enhanced the activity of mitochondrial complexes (I and II), increased the aconitase enzyme activity, boosted ATP production, and restored the levels of calcium, iron, and caspase-3, compared to the corresponding values in irradiated rats. The levels of glucose and cholesterol in brain tissue were modulated. Citicoline also increased acetylcholine level and alpha-7 nicotinic receptor mRNA expression and decreased acetylcholinesterase activity in the brain tissue of irradiated-treated rats. We concluded that citicoline could attenuate the harmful effects of γ-radiation on the brain by modulating mitochondrial function, neurotransmission, and calcium & iron homeostasis, thus suppressing the mitochondrial-mediated apoptosis pathway. However, additional studies are required to validate and confirm these results before any clinical application can be recommended.

## Introduction

In our daily lives, we may be exposed to various pollutants that may lead to many diseases, including cancer. Scientists and oncologists are always searching for effective treatments to treat cancer^1^. Radiation therapy is one of the effective therapeutic modalities established to treat cancer patients. However, short- and long-term adverse effects associated with radiation therapy require management strategies to modify these effects^2^. Head irradiation is commonly used to treat brain cancer; however, it can cause side effects in healthy brain tissue either by the direct effects of radiation or its indirect effects through the ionization of water. Since the brain is particularly susceptible to oxidative damage due to its high oxygen consumption, lipid-rich constitution, and limited antioxidant defenses^3^, whole-brain radiotherapy causes oxidative damage to brain tissue that promotes neuro-inflammation, disrupts the blood-brain barrier^4^, declines cognition^5^, and causes endocrine disturbance in hypothalamic-pituitary axis & metabolic complications^6^.

One of the main subcellular targets for oxidative damage is mitochondria. It is known to be the primary source of cellular energy through the tricarboxylic acid cycle (TCA), electron transport chain, and oxidative phosphorylation. Besides, mitochondria are responsible for regulating calcium homeostasis and metabolic activity of the cell to maintain cell survival and homeostasis. The most appropriate energy source in the brain is glucose oxidation, due to its high rate of adenosine triphosphate (ATP) generation required to maintain neuronal activity^7^. Oxidative damage to brain cells after exposure to ionizing radiation may induce mitochondrial dysfunction, leading to various pathways of cell death that may be the origin of neurodegeneration. The association between mitochondrial dysfunction and neurodegenerative diseases is common^8^. Also, an association between increased neurodegeneration risk with dyslipidemia (low high-density lipoprotein and high triglycerides) and hyperglycemia was found^9,10^. Although ionizing radiation was initially believed to primarily disrupt mitochondrial bioenergetic and biosynthetic metabolism and induce programmed cell death, it is now known that it can also influence mitochondrial epigenetic mechanisms, intracellular signaling pathways, and intercellular communication^11^.

Therefore, the tendency to use mitigating agents to alleviate the toxicity caused by exposure to ionizing radiation has attracted much attention from many researchers. It is critical to search for effective and accessible mitigating agents, especially those without any side effects^12^. Recently, the evaluation of mitochondrial functions in different diseases and designing drug therapies to target mitochondria has been pointed out by You et al.^13^.

Citicoline or cytidine diphosphate-choline is a form of choline supplement. It is a natural precursor of the neurotransmitter acetylcholine (ACh) and phospholipids, especially phosphatidylcholine. Evidence from experimental studies suggested that citicoline exerted neuroprotective and neuromodulator effects on neuronal cells, including retinal ganglion cells^14^. Citicoline is widely used in the treatment of neurodegenerative diseases such as Alzheimer’s disease^15^, serves as an adjuvant therapy for Parkinson’s disease^16^, and is also employed as an adjunct treatment to provide partial protection against experimental cerebral malaria^17^. It maintains the structural integrity of the biological membranes and enhances the synthesis of ACh^18^. Moreover, it has been shown that in the injured brain, whether due to ischemia or trauma, citicoline counteracted oxidative stress and restored phospholipid levels in cellular membranes^19–21^. Again, it was reported that citicoline positively affects memory failure in elderly individuals^22^, improves cognition in patients with mild cognitive impairment, especially of vascular origin or neurodegeneration^23^, and has clinical benefits in patients with SARS-CoV-2-induced hypoxemic acute respiratory failure^24^. Furthermore, it was observed that liposomal CDP-choline safeguarded mitochondria and restored their normal ultrastructure and functions in rats following cerebral ischemia-reperfusion^25^. In a previous study, we demonstrated that citicoline could exert modulating effects against neuroendocrine disturbances in rats exposed to head irradiation^26^. In this article, we aim to investigate for the first time (to the best of our knowledge) whether citicoline has a role in the modulation of brain mitochondrial function in male albino rats exposed to 10 Gy head irradiation, using males specifically to minimize the confounding effects of hormonal fluctuations.

## Materials and methods

### Chemicals

Citicoline was purchased from Sigma-Aldrich Co. (St. Louis, MO, USA) and dissolved in saline to obtain the necessary dose. Pentobarbital sodium salt, CAS No. 57-33-0, was also obtained from Sigma-Aldrich, USA.

### Animals

Twenty-four male Wistar rats (120–150 g) were obtained from the National Center for Radiation Research and Technology (NCRRT), Egyptian Atomic Energy Authority (EAEA), Cairo, Egypt. The animals were acclimatized for seven days and were provided with standard laboratory food and tap water ad libitum. They were kept in a temperature-controlled environment (20–25 °C) with a 12-hour dark/light cycle.

This study was approved by the Animal Ethics Committee of the National Center for Radiation Research & Technology (56 A/23). The animal procedures in this protocol were conducted in accordance with the guidelines of animal handling and care published by the National Institute of Health (NIH no. 85:23, 1985).

### Irradiation process

Gamma irradiation was performed using the Indian (^60^Co) cell at the NCRRT. The rats were anesthetized with pentobarbital (60 mg/kg (i.p.)) according to Shekarforoush et al.^27^ and then exposed to 10 Gy head irradiation (based on Abdel-Aziz et al.^26^) with a dose rate of 1.14 KGy/h, according to the Dosimetry and Protection Department in the NCRRT. However, the rest of the body was protected from radiation with a lead shield.

### Experimental design

The rats were divided into four groups, each containing six rats. Group 1 (control), the rats of this group were kept as the control. Group 2 (Citicoline), these rats were injected intraperitoneally with citicoline (300 mg/kg/d) for 4 weeks according to^26^. Group 3 (Radiation), the heads of these rats were exposed to a single dose of γ-ray (10 Gy). Group 4 (Radiation + Citicoline), rats of this group were exposed to a single dose of γ-ray as in group 3, and five minutes after irradiation, the rats were injected intraperitoneally with citicoline (300 mg/kg/d) for 4 weeks.

### Brain tissues

Four weeks after irradiation or after the last dose of citicoline, the animals were anesthetized with pentobarbital (60 mg/kg (intraperitoneal injection)) and sacrificed by decapitation. The brains were quickly dissected out. One hemisphere of the brain was used for mitochondrial isolation using a Mitochondria Isolation Kit (Catalog # MBS355483). The other brain hemisphere was kept at −80 °C until used for the other biochemical analysis.

### Biochemical analysis

Hydroxyl free radical level was determined in brain tissue using assay Kits (Cat. No. MBS2540424) following the manufacturer’s instructions. Malondialdehyde (MDA) was determined in brain mitochondria using assay Kits (Cat. No. MAK085) according to the manufacturer’s directions. Reduced glutathione (GSH) content was measured in brain mitochondria by colorimetric method using a commercial kit (CAT. No. GR 25 11) from Biodiagnostic, Egypt.

Complex I (NADH dehydrogenase), complex II (Succinate dehydrogenase), and aconitase activities were measured in brain mitochondria using colorimetric assay Kits (Cat. No. MAK 359, MAK197 and MAK051, respectively) from Sigma-Aldrich, St. Louis, Missouri, USA, according to the manufacturer’s directions. ATP was determined using a colorimetric assay Kit (Cat. No. MAK190) from Sigma-Aldrich, St. Louis, Missouri, USA. Calcium and iron levels in brain mitochondria were determined using colorimetric assay Kits (Cat. No. KA0812, Abnova Corporation; and Cat. No. E-BC-K139-S, Elabscience, respectively). Caspase-3 protein level in brain mitochondria was performed using an ELISA kit (MyBiosource, Inc., USA), catalog no. MBS700575, as described by the manufacturer.

Glucose, cholesterol, triglyceride, LDL-C, HDL-C, and protein levels were measured in brain tissue using colorimetric assay kits (MyBiosource, Inc., USA) following the manufacturer’s instructions. The corresponding catalog numbers were: MBS8243232 (glucose), MBS168179 (cholesterol), MBS168769 (triglycerides), MBS2540573 (LDL-C), MBS2540578 (HDL-C), and MBS2540542 (protein).

Acetylcholine was determined in brain tissue using EnzyChromTM Acetylcholine Assay Kit, Cat. No: EACL-100 (BioAssay Systems), according to the manufacturer’s directions. Acetylcholinesterase (AChE) activity was measured using a Rat ELISA Kit (E-BC-K174-M), Elabscience Biotechnology Inc. The level of alpha-7 nicotinic receptor (α7 nAChR) gene expression was measured in brain tissue using a quantitative real-time PCR assay with SYBR Green. The relative expression of α7 nAChR was calculated according to Applied Biosystem software using the comparative threshold cycle method^28^. The values were normalized to the beta-actin expression (reference gene).

### Primer sequences for quantitative RT-PCR analysis

α7nAChR (Gen Bank accession number): NM_012832.

F: 5′-GCAAAGAGCCATACCCAG-3′.

R: 5′-CAGCAAGAATACCAGCAGAG-3′.

b-actin (Gen-Bank accession number): NM_031144.

F: 5’-TTGTCCCTGTATGCCTCT-3′.

R: 5’-TAATGTCACGCACGATTTCC-3′.

### Statistical analysis

Statistical analysis was performed using the SPSS program (version 20). The values were presented as mean ± SD. The data were analyzed using a one-way analysis of variance followed by the least significant difference post hoc test to determine significant differences between means. The significance levels were set at *p* < 0.05.

## Results

**Fig. 1:**
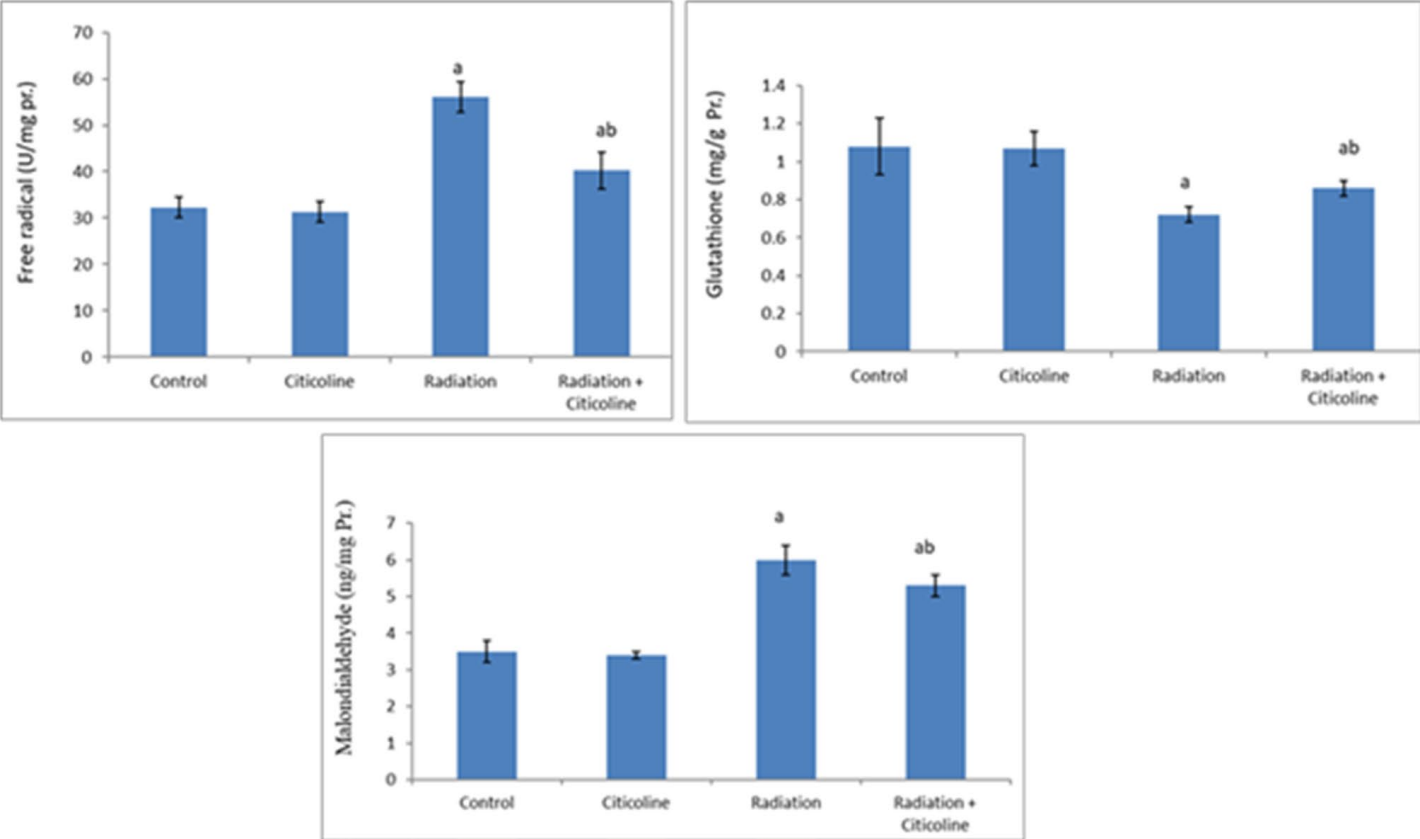
Free radicals in rat brain tissue and Malondialdehyde and glutathione levels in rat brain mitochondria following head irradiation and/or citicoline administration. Data are expressed as means ± standard deviation (*n*=6). ^a^: Significance versus Control. ^b^: Significance versus Radiation. The mean difference is significant at the 0.05 level.

**Fig. 2:**
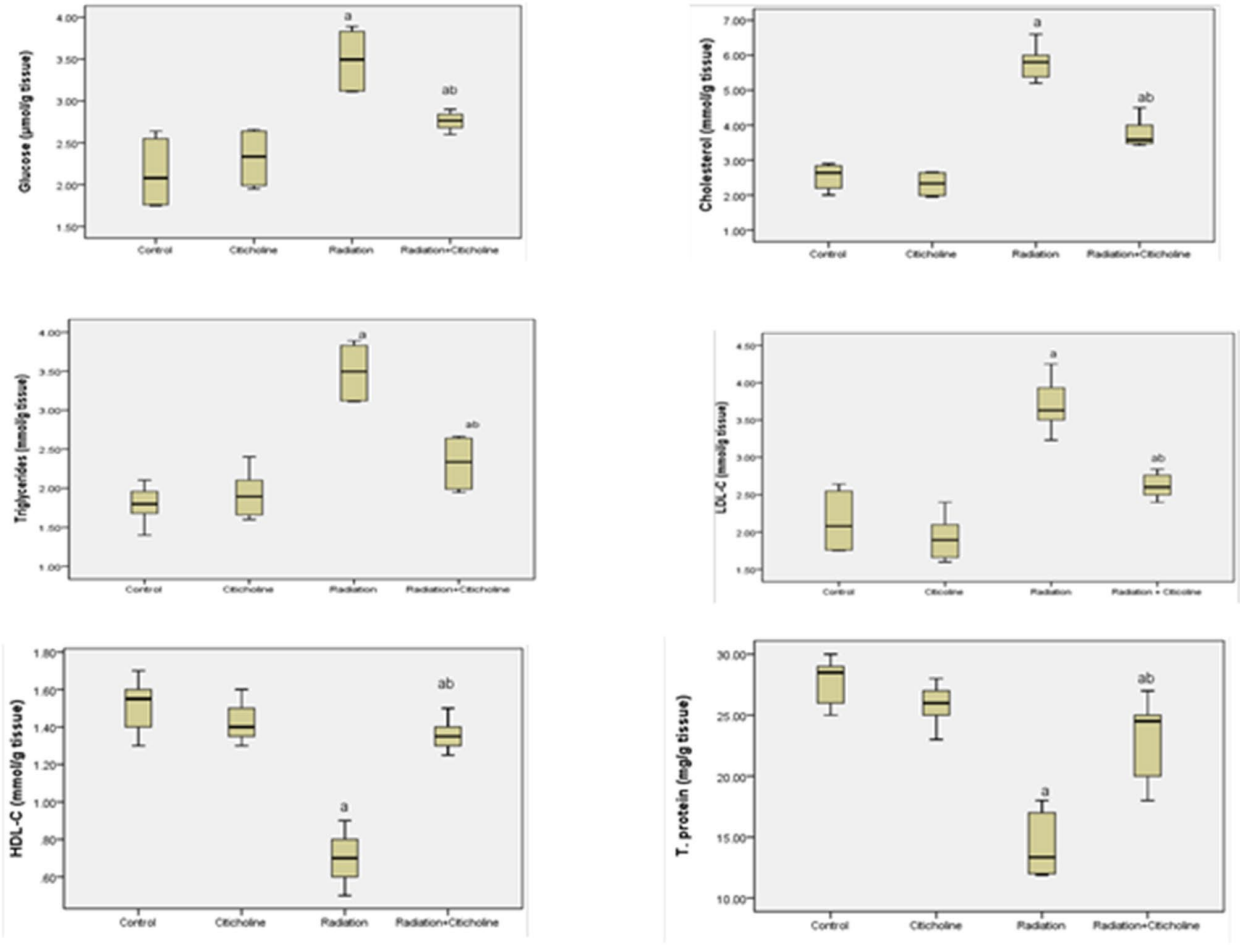
Glucose, Cholesterol, Triglycerides, low-density lipoprotein (LDL-C), high-density lipoprotein (HDL-C), and Total protein levels in rat brain tissue following head irradiation and/or citicoline administration. ^a^: Significance versus Control. ^b^: Significance versus Radiation. The mean difference is significant at the 0.05 level.

**Fig. 3:**
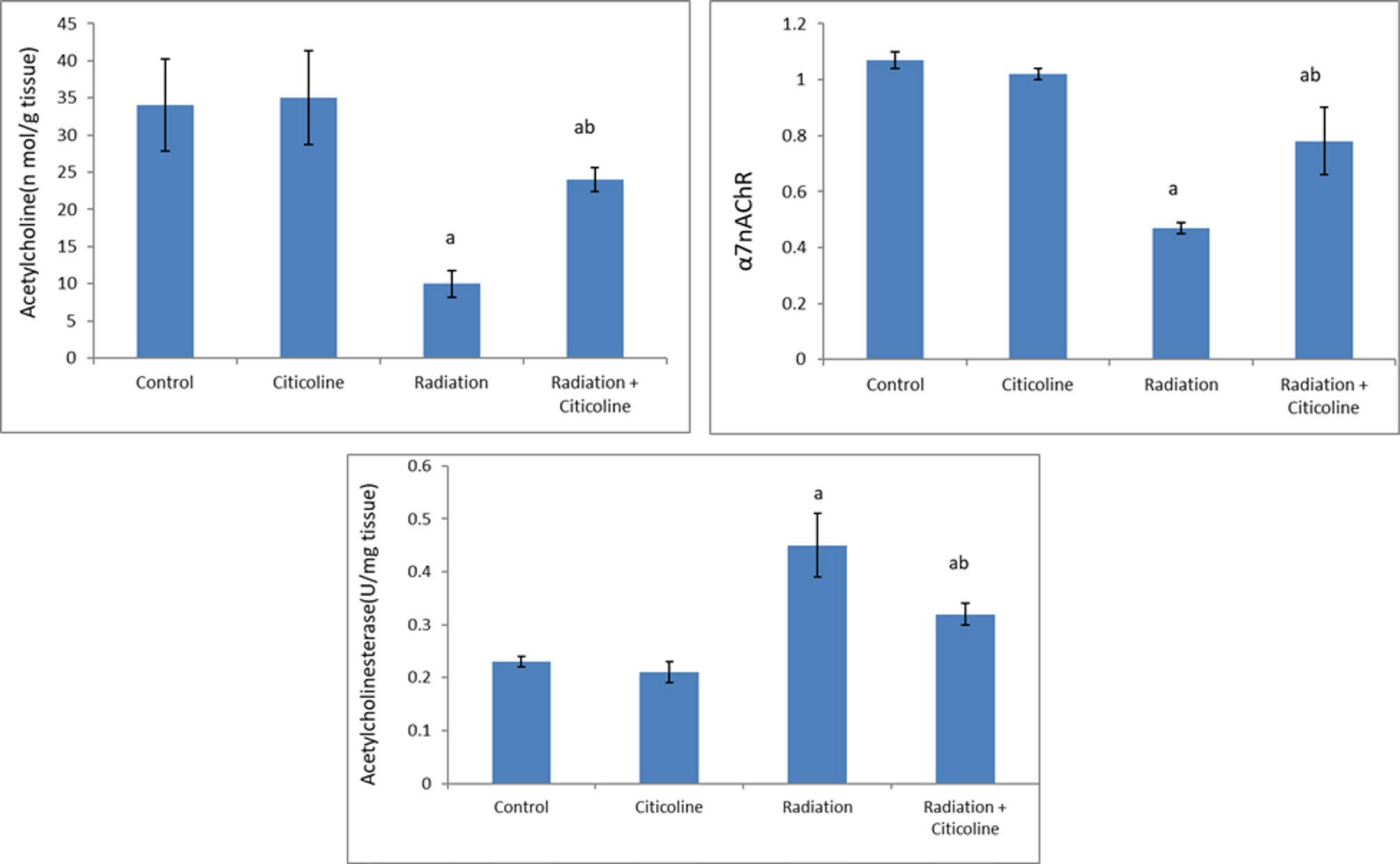
Acetylcholine level, alpha7nicotinic acetylcholine receptor (α7nAChR) mRNA expression, and Acetylcholinesterase activity in rat brain tissue following head irradiation and/or citicoline administration. Data are expressed as means ± standard deviation (*n* = 6). ^a^: Significance versus control. ^b^: Significance versus Radiation. The mean difference is significant at the 0.05 level.

Biochemical Effects of Head Irradiation on the Brain:

The results of the current study showed that 10 Gy head irradiation induced a marked increase (*p* < 0.05) in free radicals’ levels in brain tissue and MDA level in brain mitochondria associated with a significant decrease (*p* < 0.05) in mitochondrial glutathione level (Fig. 1) compared to their values in the control group. In addition, head irradiation resulted in a significant drop (*p* < 0.05) in the activities of mitochondrial complex I, complex II & aconitase enzyme, and ATP production (Table 1), as well as a marked rise (*p* < 0.05) in calcium, iron, and caspase 3 levels, compared to their control levels (Table 2). Furthermore, irradiation led to a significant increase (*p* < 0.05) in glucose, cholesterol, TG, and LDL-C levels associated with a significant drop (*p* < 0.05) in HDL-C and protein levels in brain tissue (Fig. 2). A marked decrease (*p* < 0.05) in ACh level and the expression level of α7nAChR along with a marked increase (*p* < 0.05) in AChE activity was observed in the irradiated group compared to the control group (Fig. 3).

**Table 1:** Complex I, complex II, and aconitase activities and ATP level in rat brain mitochondria following head irradiation and/or citicoline administration.

Parameters Groups	Complex l (mU/mg Pr.)	Complex П (mU/mg Pr.)	Aconitase (mU/mg Pr.)	ATP (nmol/mg Pr.)
**Control**	3.3 ± 0.3	2.2 ± 0.3	4.5 ± 0.3	3.3 ± 0.4
**Citicoline**	3.5 ± 0.1 (+ 6)	2.1 ± 0.3 (−4.5)	4.3 ± 0.2 (−4%)	2.9 ± 0.2 (−12)
**Radiation**	2.1 ± 0.4 (−36%) ^a^	1.3 ± 0.1 (−41%) ^a^	2.1 ± 0.4 (−53%) ^a^	1.3 ± 0.1 (−61%) ^a^
**Radiation + Citicoline**	2.7 ± 0.1 (−18%) ^ab^	1.8 ± 0.2 (−18%) ^ab^	3.5 ± 0.3 (−22%) ^ab^	2.1 ± 0.3 (−36%) ^ab^

Data are expressed as means ± standard deviation (*n* = 6). The number in brackets shows the percentage of change from the respective control value. ^a^: Significance versus Control. ^b^: Significance versus Radiation. The mean difference is significant at the 0.05 level.

**Table 2:** Calcium, iron, and Caspase-3 levels in rat brain mitochondria following head irradiation and/or citicoline administration.

Parameters Groups	Calcium (mg/g tissue)	Iron (µmol/g tissue)	Caspase-3 (ng/g tissue)
**Control**	1.5 ± 0.5	3.4 ± 0.1	32 ± 5.1
**Citicoline**	1.7 ± 0.4 (+ 13%)	3.5 ± 0.1 (+ 3%)	30 ± 5.5 (−6%)
**Radiation**	2.5 ± 0.4 (+ 67%) ^a^	6.4 ± 0.4 (+ 88%) ^a^	56 ± 3.1 (+ 75%) ^a^
**Radiation + Citicoline**	1.9 ± 0.2 (+ 27%) ^b^	5.2 ± 0.1 (+ 52%) ^ab^	45 ± 6.2 (+ 41%) ^ab^

Data are expressed as means ± standard deviation (*n* = 6). The number in brackets shows the percentage of change from the respective Control value. ^a^: Significance versus Control. ^b^: Significance versus Radiation. The mean difference is significant at the 0.05 level.

Biochemical Effects of Citicoline Administration on the Brain:

Citicoline administration to normal rats indicated non-significant changes in the studied parameters compared to their normal control counterparts (Tables 1 and 2; Figs. 1, 2 and 3). However, Citicoline administration after head irradiation resulted in a marked decrease in free radical and MDA levels associated with a significant increase (*p* < 0.05) in glutathione level (Fig. 1), a marked increase (*p* < 0.05) in the activities of mitochondrial Complex I, complex II & aconitase enzyme, and ATP production (Table 1), besides a marked decrease (*p* < 0.05) in calcium, iron, and caspase 3 levels (Table 2) in mitochondria, compared to irradiated-non treated rats. In addition, rats treated with citicoline after irradiation showed a significant decrease (*p* < 0.05) in glucose, cholesterol, TG, and LDL-C levels associated with a significant increase (*p* < 0.05) in HDL-C and protein levels in brain tissue compared to the irradiated group (Fig. 2). A noticeable increase (*p* < 0.05) in ACh level and the expression level of α7nAChR associated with a significant decrease (*p* < 0.05) in AChE activity was observed in brain tissue of the irradiated-treated group, relative to the irradiated group (Fig. 3).

## Discussion

Head irradiation is widely used in the treatment of brain tumors. However, its side effects significantly impact patients’ quality of life. Our previous study revealed neuroendocrine disruptions in the brain tissue of rats subjected to head irradiation. We also observed that citicoline can exert modulating effects against these disturbances through the modulation of neurotransmitters and pituitary circulating hormones^26^. However, its effect on mitochondrial function in irradiated rats is still missing. Therefore, in this article, we aimed to investigate the impact of citicoline on the brain mitochondrial function and metabolic sequelae in rats exposed to 10 Gy head irradiation.

Mitochondria are known to be the main source of endogenous cellular reactive oxygen species (ROS). Although modest levels of ROS stimulate essential biological processes, many disorders in mitochondrial components and, consequently, mitochondrial function have been linked to high concentrations of free radicals^29^. It has been reported that excess ROS, induced by exposure to ionizing radiation, may not only disrupt mitochondrial function and cellular bioenergetic status, but also cause mutations in mitochondrial DNA and genomic instability^11^. Since mitochondria play a vital role in numerous neural functions- including neuronal growth and development, synaptic signaling, and the preservation of neuronal plasticity and connectivity- mitochondrial dysfunction can lead to disruptions in energy metabolism and cellular death, ultimately resulting in central nervous system dysfunction. This dysfunction may be a key trigger for neurodegenerative diseases^30^. Therapeutic strategies aimed at reducing oxidative stress and modulating mitochondrial dysfunction- through boosting antioxidant defenses, enhancing mitochondrial biogenesis, and improving mitochondrial dynamics- may hold significant promise for treating neurodegenerative diseases or slowing their progression^31,32^.

The present investigation revealed that exposure of rats to 10 Gy head irradiation triggered oxidative stress, indicated by a significant increase in MDA levels associated with a significant decrease in GSH content in brain mitochondria and a significant increase in free radicals in brain tissue. This effect may be due to the interaction of ROS generated after irradiation with cellular and subcellular macromolecules, producing harmful free radicals leading to lipid peroxidation and a decline in the endogenous antioxidants^26,33^. Several studies indicated that the imbalance between free radical production and antioxidant defenses increases the concentrations of these radicals and their consequent action on biological molecules, resulting in pathological implications, including mitochondrial and metabolic disturbances^11,34^. The oxidative stress observed in this study was associated with a significant increase in brain glucose, cholesterol, triglyceride, and LDL-C levels, and a significant decrease in HDL-C and protein levels. Glucose oxidation is the primary source of energy in the brain. Inhibition of brain glucose metabolism reduced energy production and thus impaired neuronal function^7^. These may lead to neuroendocrine disorders (decrease in neurotransmitters and dysregulation in pituitary hormones) that induce disturbances in various metabolic pathways^26^. Indeed, cranial irradiation induces hypothalamic-pituitary disorders, including growth hormone deficiency^35^, leading to an increase in total cholesterol, triglyceride, and LDL-C levels and a decrease in HDL-C levels. However, growth hormone replacement therapy has been shown to improve lipid profile^36^, activate neural stem cells, and elevate neurogenesis^37^.

Again, the imbalance between free radicals and GSH levels observed in this study is associated with an impairment of brain mitochondrial function represented by a significant decrease in the mitochondrial complexes I & II, aconitase enzyme activity, and ATP production. Comparable results have been recorded in the previous study of Abdel-Magied et al.^38^ using 7 Gy whole-body radiation. These results indicated that mitochondria are a target of radiation toxicity that inhibits the metabolic pathway of oxidative phosphorylation responsible for ATP production. Moreover, the oxidative damage induced by exposure to ionizing radiation increases the proteolytic susceptibility of mitochondrial aconitase, an enzyme involved in the regulation of the TCA cycle^39^. The inhibition of the TCA cycle reduces glucose metabolism and directs citrate (which can cross the mitochondrial membrane and enter the cytoplasm) to the biosynthesis of fatty acids, triglycerides, and cholesterol due to damage to the aconitase enzyme, which is responsible for converting citrate to isocitrate^40^. So, it is suggested that the reduction of glucose oxidation may also result from oxidative damage to the Fe–S clusters of the aconitase enzyme. It has been reported that the Fe–S clusters in aconitase can be disassembled upon exposure to various oxidants^40^, and the release of reactive iron may further induce oxidative damage to cellular components. In addition, oxidative stress may dysregulate calcium homeostasis and alter mitochondrial membranes^41^. The results of this study fall in the same line that head irradiation induced a significant increase in iron and calcium levels. When calcium ions are overloaded in the mitochondria, there is a consequent increase in ROS production, a decrease in ATP synthesis, a change in mitochondrial membrane permeability, a release of cytochrome c, and activation of caspases and apoptosis^42^.

The increase in mitochondrial membrane permeability may lead to cholinergic neuronal death resulting in a reduction in ACh synthesis. Besides this, exposure to ionizing radiation increases the activity of acetylcholinesterase (AChE), the enzyme responsible for the breakdown of ACh^43^. In this context, it has been reported that ACh or choline (a precursor of ACh) prevents Ca^2+^ accumulation in mitochondria. Similarly, ACh attenuates the release of ROS and cytochrome c from the mitochondria^44,45^.

Therefore, we thought of investigating the effect of citicoline in restoring mitochondrial function in the brains of rats exposed to head irradiation, where it is considered an ACh donor and phosphatidylcholine (one of the most abundant phospholipids of the mitochondrial membranes that contribute to membrane structure and function) precursor.

It has been reported that in the injured brain, citicoline counteracts oxidative stress and maintains the structural integrity and signaling functions of mitochondrial membranes by restoring the phospholipids (phosphatidylcholine, cardiolipin & sphingomyelin) levels in mitochondrial membranes that are vital for mitochondrial function^19–21^. Our results fall in the same line that treatment of γ-irradiated rats with citicoline inhibited the formation of free radicals & MDA and increased the level of GSH compared with those of the irradiated group. These results agree with a previous study, indicating that citicoline provided neuroprotection by increasing GSH synthesis & superoxide dismutase activity and decreasing MDA & nitrite levels against ischemic brain injury^19^. Previously, it has been reported that the choline liberated from citicoline can be metabolized to a betaine which is metabolized further to GSH^46^. Moreover, citicoline restored mitochondrial oxidative damage by increasing the GSH/GSSG ratio and decreasing lipid peroxidation against ischemic liver injury^47^. The latter hypothesized that the antioxidant effect of citicoline may result from the stabilization of biological membranes by the synthesis of phosphatidylcholine. The antioxidant and antiaging activities of phosphatidylcholine have been reported by Kim et al.^48^.

Citicoline administration to irradiated rats ameliorated the levels of glucose, cholesterol, triglycerides, and protein, compared with those of the irradiated group. This result agrees with the earlier study of Kakihana et al.^49^, it has been denoted that citicoline modulated the disruption of cerebral glucose metabolism in an animal model of cerebral ischemia. In addition, since choline is essential for the synthesis of phospholipids, it could prevent excessive lipid accumulation^50^ and improve lipid profiles and cardiovascular health^51^.

The results also showed that citicoline administration increased the activities of mitochondrial enzyme complexes (I and II) & aconitase enzyme with an increase in ATP production and a decrease in iron, calcium, and caspase 3 levels compared with those of the irradiated group. These results are consistent with previous demonstrations that citicoline preserved the activity of the aconitase enzyme and maintained mitochondrial calcium levels in the hearts of hyperthyroid rats^52^. Again, citicoline improved mitochondrial viability and maintained mitochondrial enzyme complexes (I, II, and IV) & aconitase activity in different ischemic brain tissues^19,47^. Additionally, it was demonstrated that citicoline can preserve the cardiolipin level required for Fe-S cluster biogenesis and the maintenance of mitochondrial and cellular iron homeostasis^53,54^. Supporting our results, previous studies indicated that citicoline enhances the activity of lipid-dependent enzymes of the TCA cycle and the mitochondrial respiratory chain in patients with chronic cerebral ischemia^55^ and restores the oxidative phosphorylation in influenza A virus-infected mice^56^. In the same context, liposomal CDP-Choline has been observed to protect brain cell mitochondria from injury arising from cerebral ischemia-reperfusion in both young and aged rats. It exhibits an integrated mitochondrial ultrastructure with improved cristae and matrix configuration^25^. The study by Zhong et al. showed that citicoline protected against neomycin-induced injury by increasing mitochondrial membrane potential and reducing apoptosis in HEI-OC-1 cells (House Ear Institute-organ of Corti 1)^57^. These demonstrations support our hypothesis that citicoline can preserve brain mitochondrial function in irradiated rats.

It can be concluded that citicoline could attenuate the harmful effects of γ-radiation on the brain by modulating mitochondrial function, neurotransmission, and calcium & iron homeostasis, thus suppressing the mitochondrial-mediated apoptosis pathway. However, additional studies are necessary to validate these findings before a clinical application can be recommended.

## Data Availability

The data used in the current study are included in the published article.

## Abbreviations

Ach: Acetylcholine
AChE: Acetylcholinesterase
ATP: Adenosine triphosphate
MDA: Malondialdehyde
GSH: Reduced glutathione
ROS: Reactive oxygen species
TCA: Tricarboxylic acid cycle
α7 nAChR: Alpha-7 nicotinic receptor
